# Exploring the Link Between Age Segregation and Loneliness Among Older Adults

**DOI:** 10.1093/geroni/igaf122.1129

**Published:** 2025-12-31

**Authors:** Seulki Kim, Seung-won Emily Choi

**Affiliations:** University of Nebraska-Lincoln, Lincoln, Nebraska, United States; Texas Tech University, Lubbock, Texas, United States

## Abstract

Residential segregation has been associated with various mental and physical health outcomes; however, the segregation of different age groups has garnered limited attention until recently, and its impact on health remains underexplored. Specifically, there is little knowledge about how living in age-segregated communities influences feelings of loneliness in later life. Drawing on both individual and geographic data from the Health and Retirement Study (2010-2018) and the American Community Survey, this study is among the first to explore the relationship between age segregation and loneliness among older adults, with a particular focus on gender differences. Our analytic sample includes 5,282 adults aged 65 and older in 2010, all of whom resided in the same neighborhoods throughout the study period. Results from hierarchical linear modeling indicate that individuals living in neighborhoods with higher age segregation report lower levels of loneliness. Notably, this protective effect is observed only among older women. The robust link between age segregation and loneliness highlights the importance of considering age-based residential segregation as a key neighborhood factor affecting the mental health in later life. We suggest that interventions aimed at addressing mental health disparities among older adults in age-segregated community settings consider gender differences.

